# Effects of permanent placental injury due to severe acute respiratory syndrome coronavirus 2 infection during pregnancy on the feto-placental circulation: a cross-sectional study

**DOI:** 10.1590/1806-9282.20230827

**Published:** 2024-02-26

**Authors:** Mihriban Kılçar, Ümran Kılınçdemir Turgut, Kemal Kürşat Bozkurt, Gökhan Bayhan

**Affiliations:** 1 Süleyman Demirel University, Faculty of Medicine, Department of Obstetrics and Gynecology - Isparta, Turkey.; 2 Süleyman Demirel University, Faculty of Medicine, Department of Pathology - Isparta, Turkey.

**Keywords:** Ultrasonography, doppler. Pregnancy. Placenta. SARS-CoV-2. Ultrasonography

## Abstract

**OBJECTIVE:**

The aim of this study was to evaluate the effects of permanent placental injury due to a severe acute respiratory syndrome coronavirus 2 infection during pregnancy on feto-placental circulation.

**METHODS:**

In this cross-sectional study, 83 pregnant women with planned deliveries were divided into two groups according to their severe acute respiratory syndrome coronavirus 2 infection statuses during pregnancy. Their demographic parameters, obstetric histories, and prenatal risks were evaluated. A prenatal fetal Doppler ultrasound examination was performed for all participants, and umbilical artery and middle cerebral artery Doppler parameters were obtained. Postpartum placentas were examined for pathological findings under appropriate conditions. All placentas were evaluated according to the Amsterdam consensus criteria. Mann-Whitney U test, Student’s t-test, and chi-square test were used for comparisons.

**RESULTS:**

Demographic parameters were statistically similar, except that they were borderline significant for gestational weeks at delivery (p=0.044). In the pathological examination of the placenta, regardless of the trimester of exposure to viral infection, perivillous fibrin deposition and villus dystrophic calcification were more common in group 2 (p=0.016 and p=0.048, respectively) than in group 1. In the prenatal Doppler examination between the groups, no statistically significant difference was found for all of the umbilical artery pulsatile index, middle cerebral artery pulsatile index, and cerebro-placental ratio values.

**CONCLUSION:**

Severe acute respiratory syndrome coronavirus 2 infection during pregnancy causes an increase in perivillous fibrin deposition and villus dystrophic calcification in the placenta. Placental injury caused by the severe acute respiratory syndrome coronavirus 2 virus does not affect fetal Doppler parameters.

## INTRODUCTION

The severe acute respiratory syndrome coronavirus 2 (SARS-CoV-2) infection, which was declared a pandemic by the World Health Organization, continues to be an important health problem. This novel coronavirus has caused over 6.5 million deaths worldwide as of mid-2023^1^.

According to the National Institutes of Health (NIH), SARS-CoV-2 infection can be classified into five categories based on the disease symptoms and clinical findings: asymptomatic or presymptomatic infection, mild illness, moderate illness, severe illness, and critical illness^2^.

Some scientists have suggested that this virus may adversely affect the placenta and cause fetal infection by transmitting the SARS-CoV-2 receptor-due to angiotensin-converting enzyme 2-which is abundant in the human placenta^3^.

Placental infection has been defined by demonstrating SARS-CoV-2 in chorionic villous cells using the nucleic acid methods such as immunohistochemistry and in situ hybridization^4^. However, the vertical transmission of this novel coronavirus from mother to fetus has been demonstrated to be very low^5^. The prevalence of decidual arteriopathy and maternal vascular malperfusion (MVM) has been shown to increase when the pathological evaluation of the placenta is conducted in pregnant women with SARS-CoV-2 infection^6^. In a recently published review, placental hypoperfusion and inflammation, including perivillous fibrin deposition, have been found to be more common in the placentas of pregnant women infected with SARS-CoV-2 than those of noninfected pregnant women^7^.

The effects of pathological damage in the placenta on fetal Doppler parameters are known.

In the past, the relationship between lesions in various placental pathological examinations and fetal Doppler parameters has been described^8^ ^,^ ^9^. Although many studies have examined placental injury during acute viral infection, those that investigate the status of the placenta after its previous infection are limited. In addition, more studies are needed on the effects of this injury on feto-placental circulation.

The aim of this cross-sectional study was to evaluate the effects of pathological placental injury and its reflections on fetal Doppler examination parameters in pregnant women who had SARS-CoV-2 infection during pregnancy.

## METHODS

The study protocol was approved by the local ethics committee (13.07.2021/237). Informed consent was obtained from each participant. Pregnant women with singleton gestation who presented to the emergency department due to labor onset or a planned elective caesarean section between July 2021 and September 2021 were included in this cross-sectional study.

The control group (group 1) included pregnant women who did not have a proven SARS-CoV-2 infection or did not show symptoms of the disease during pregnancy and did not have any prenatal fetal risk factors. The patient group (group 2) included pregnant women who had SARS-CoV-2 infection proven by the nasal and throat swab samples at 4 weeks of gestation or later but had negative polymerase chain reaction (PCR) results for SARS-CoV-2 in the nasal and throat swab samples at delivery.

The exclusion criteria for both groups were chronic hypertensive disease, pre-eclampsia/eclampsia, multiple pregnancies, clinical or laboratory findings of chorioamnionitis, placental abruption, the presence of other active viral or bacterial infection, the presence of fetal structural or genetic anomaly, placenta previa, placenta accreta suspicion, symptoms of viral infection, and SARS-CoV-2 PCR positivity in the nasal and throat swab samples at delivery.

Demographic parameters, obstetric histories, and perinatal risks [i.e., gestational week, gravity-parity-abortion number, disease history (e.g., diabetes mellitus, hypothyroidism), gestational diabetes mellitus (GDM), gestational hypertension, intrauterine growth restriction, gestational cholestasis, small gestational age (SGA), and preterm premature rupture of membranes (PPROM)] were obtained for all the included participants. Information about the gestational week when the patient was diagnosed with a positive PCR result and their symptoms during infection was also obtained from the patients in group 2.

A prenatal Doppler examination was conducted for the participants who met the inclusion and exclusion criteria. The ultrasound scans were obtained with the Voluson E6 Ultrasound System (GE Healthcare, Little Chalfont, UK) using a convex 1e5-MHz probe.

All prenatal Doppler examinations were evaluated by the same perinatology specialist (ÜKT), without knowledge on which group the pregnant women belonged to (single blind). Umbilical artery Doppler examination was performed on the free cord while the fetus was immobile. MCA Doppler was performed from the proximal segment when the insonation angle was between 0° and 15°. Umbilical artery pulsatile index (PI); resistance index (RI) and systole/diastole (S/D) values; middle cerebral artery (MCA) PI, RI, and S/D values; and cerebro-placental ratio (CPR) values calculated on our ultrasound imaging system (ViewPoint6, GE Healthcare, Chicago, IL, USA) were obtained.

The placenta was preserved for pathological examination under appropriate conditions after delivery. A pathological examination was performed by the same pathologist (KKB), without knowledge on which group the pregnant women belonged to (single blind). Placental tissue was preserved in 10% formol, and placental weights were measured after macroscopic examination. Notably, 5-µm-thick preparations obtained from paraffin blocks were stained with hematoxylin and eosin. The preparations were evaluated with light microscopy. The placentas were examined using a standardized methodology, the Amsterdam consensus criteria.

### Statistical analysis

The data were evaluated using statistical analysis. The means and standard deviations were given for the descriptive statistics of continuous variables, and the number of people (n) and percentages (%) were given for the definitions of categorical variables. The relationships between categorical variables were examined using chi-square test analysis. Whether the continuous variables showed a normal distribution was checked with the Shapiro-Wilk test of normality, and homogeneity of variance was checked with the Levene test. Bi-level comparisons were conducted using the Mann-Whitney U test in cases in which there was no normal distribution and with the t-test when assumptions were met.

## RESULTS

A total of 83 pregnant women who met the criteria were included in this cross-sectional study. While 44 pregnant women were included in group 1, 39 pregnant women were infected with SARS-CoV-2 during their pregnancy and were included in group 2.

All the demographic parameters (maternal age, body mass index, gravity, parity, and abortion rates) were statistically similar, except that they were borderline significant for gestational weeks at delivery (p=0.044) (Table 1). The prevalence of GDM and hypothyroidism was similar between the groups. There were 3 (7.8%) SGA cases, 5 (12.8%) PPROM cases, and 1 (2.6%) polyhydramnios case in group 2.

**Table 1.** Demographic parameters.

	Group 1 (n=44)	Group 2 (n=39)	p
	Mean±standard deviation		
Maternal age (years)	30.41±5.95	28.85±5.16	0.208
BMI	29.26±5.36	29.26±3.98	0.745
Gestational weeks	38.48±1.16	37.47±2.29	0.044*
Gravity	2.75±1.45	2.64±1.89	0.375
Parity	1.30±1.27	1.13±1.13	0.645
Abortion	0.48±0.73	0.53±1.25	0.413

*p<0.05; BMI: body mass index. Mann Whitney-U test, T-test.

However, 7 pregnant women in the first trimester, 16 pregnant women in the second trimester, and 15 pregnant women in the third trimester were infected with SARS-CoV-2 in group 2. One pregnant woman could not remember the exact gestational week of the viral infection. In addition, according to the NIH scoring, 8 patients were asymptomatic, 29 pregnant women had mild disease symptoms, and 2 patients had moderate disease symptoms.

Placental findings between the groups, which were examined according to the Amsterdam consensus criteria, were evaluated. In the pathological examination of the placentas, regardless of the trimester of exposure to viral infection, perivillous fibrin deposition and villus dystrophic calcification were found to be more common in group 2 (p=0.016 and p=0.048, respectively) than in group 1. In the control group, a statistically significant increase in syncytial knotting was more frequent. There was no placenta with abnormal pathological findings in any of the fetal vascular malperfusion (FVM) examinations except for corangiosis. However, corangiosis was not statistically different between the groups. Other findings using the Amsterdam consensus criteria were found to be similar between the groups (Table 2).

In the prenatal Doppler examination between the groups, no statistically significant difference was found between the two groups for all the umbilical artery PI, MCA PI, and CPR values (Table 3).

**Table 2.** Comparison of placental pathological findings according to groups.

Placental findings	Group 1 (n=44)	Group 2 (n=39)	p
Placental weight (g)	533.75±197.18	505.14±146.73	0.989
	n (%)		
Maternal vascular malperfusion (MVM)			
Villous infarction	7 (15.9)	2 (5.1)	0.163
Distal villous hypoplasia	19 (43.2)	14 (35.9)	0.499
Increased syncytial knotting	22 (50.0)	11 (28.2)	0.043*
Maternal decidual arteriopathy	1 (2.3)	4 (10.3)	0.182
Thrombus formation in stem villi	2 (4.5)	4 (10.3)	0.316
Fetal vascular malperfusion (FVM)			
Avascular fibrotic villi	0 (0.0)	0 (0.0)	NS
Stem villous vascular obliteration	0 (0.0)	0 (0.0)	NS
Thrombosis	0 (0.0)	0 (0.0)	NS
High-grade fetal vascular malperfusion	0 (0.0)	0 (0.0)	NS
Chorangiosis	1 (2.3)	1 (2.6)	0.999
Intramural fibrin deposition	0 (0.0)	0 (0.0)	NS
Villous stromal-vascular karyorrhexis	0 (0.0)	0 (0.0)	NS
Additional findings			
Perivillous fibrin deposition	24 (54.5)	31 (79.5)	0.016*
Chorioamnionitis	1 (2.3)	2 (5.1)	0.598
Villus dystrophic calcification	1 (2.3)	6 (15.4)	0.048*
Other	1 (2.3)	2 (5.1)	0.598

*p<0.05; NS: not significant. Chi-square test.

**Table 3.** Comparison of middle cerebral artery and umbilical artery Doppler parameters according to groups.

	Group 1 (n=44)	Group 2 (n=39)	p
	Mean±standard deviation		
MCA S/D	96.76±609.99	9.72±29.37	0.605
MCA PI	1.55±0.51	1.62±0.49	0.602
MCA PI Z-score	-0.35±2.18	-1.90±17.62	0.278
MCA PI percent	43.62±36.75	38.30±35.01	0.481
MCA RI	0.75±0.12	0.76±0.10	0.579
Umbilical S/D	2.43±0.60	2.38±0.49	0.989
Umbilical PI	0.87±0.18	0.86±0.19	0.656
Umbilical PI Z-score	-0.03±1.14	-0.24±1.30	0.449
Umbilical PI percent	48.37±32.82	44.30±32.10	0.534
Umbilical RI	2.79±14.69	0.56±0.08	0.699
CPR	1.82±0.61	1.82±0.67	0.963
CPR Z-score	-0.22±1.49	-0.29±1.67	0.954
CPR percent	48.58±48.78	48.78±37.78	0.942

MCA: middle cerebral artery; S/D: systole/diastole; PI: pulsatile index; RI: resistance index; CPR: cerebro-placental ratio. Mann Whitney-U test, T-test.

## DISCUSSION

In this study, perivillous fibrin deposition and villus dystrophic calcification were found to be significantly higher in the placentas of SARS-CoV-2-infected women than in those in the control group. Although the increased syncytial knotting in the MVM category was significantly higher in the control group, it was thought to be a statistical coincidence. However, there was no significant difference between the MVM and FVM categories. No reflection of increased placental injury due to SARS-CoV-2 viral infection during pregnancy was observed in feto-placental circulation, and no significant difference was found between the groups in umbilical artery and MCA Doppler parameters.

Jaiswal et al., found in their research, which included 27 pregnant women with SARS-CoV-2 PCR+detected in nasal swab samples, that the placentas of infected cases had significantly higher MVM and FVM but that these pathological findings were not associated with poor perinatal outcomes^10^. In contrast, Levitan et al., examined the placentas of 65 pregnant women infected with SARS-CoV-2 and determined the overall prevalence of MVM to be similar when the cases were compared to the controls^11^. A review showed that placental hypoperfusion and inflammation findings, including perivillous fibrin deposition, were more common in the placentas of pregnant women infected with SARS-CoV-2 and that no increase was found in MVM while an increase was found in FVM in the same study^7^. The placentas of pregnant women infected with SARS-CoV-2 were examined according to the Amsterdam criteria, and only perivillous fibrin deposition as a pathological finding was found to be higher than those in the control group^7^. The delivery occurred during an acute viral infection, and the placenta was examined in all of these studies. However, the permanent findings in the placenta may actually be different after the viral infection has recovered. In a recent study, the researchers showed that the pathological findings were different in placentas obtained during acute and subacute viral infections. The prevalence of FVM in acute SARS-CoV-2 infection was higher than that in subacute viral infection^12^. In our study, perivillous fibrin deposition and villus dystrophic calcification findings were persistent even after the infection had recovered.

The effects of pathological damage in the placenta on fetal Doppler parameters are known. It was determined that superficial implantation and maternal hypoperfusion were present in the placentas of fetuses with abnormal umbilical artery Doppler parameters^8^. Salafia et al., showed an increase in severe villous fibrosis and intraplacental vaso-occlusive lesions in the placentas of fetuses with impaired umbilical artery flow parameters^9^. Curtis et al., found that abnormal Doppler parameters in the umbilical artery and MCA were associated with more placental pathology than those of fetuses that had normal Doppler results. In addition, abnormal MCA Doppler had a higher positive predictive value in predicting placental injury^13^. In our study, the effect of permanent damage in the placenta due to viral infection on feto-placental circulation and fetal Doppler parameters could not be demonstrated. Consistent with our study, the literature also shows that feto-placental circulation is not affected during acute SARS-CoV-2 infection^14^ ^,^ ^15^. Many studies have investigated placental pathological status and fetal Doppler parameters during acute viral infection. Knowledge of fetal and placental effects after recovering from the SARS-CoV-2 infection is limited. The present study showed that although SARS-CoV-2 viral infection during pregnancy causes placental damage pathologically, it does not cause deterioration in fetal Doppler parameters.

One limitation of this study is the possibility of including asymptomatic pregnant women in the control group. Patients with asymptomatic viral infection may not have been tested and they do not know they have been infected. Considering that pregnant women were exposed to the disease earlier than the study date, the prevalence of SARS-CoV-2 viral infection was lower in our city. In addition, asymptomatic patients were tested for the purpose of filiation. Therefore, the probability of inclusion of an asymptomatic pregnant woman in group 1 is low. Another limitation is that there were no pregnant women in group 2 with severe and critical viral infection symptoms. One reason for this is the small number of patients presenting with severe and critical symptoms in the population. Another reason is the iatrogenic or obstetric delivery of pregnant women with severe disease symptoms. Finally, in group 2, a small number of pregnant women with prenatal fetal risk were included. However, the effects of these defined prenatal risks on the placenta or fetal Doppler were not expected risks.

In conclusion, SARS-CoV-2 infection during pregnancy causes an increase in perivillous fibrin deposition and villus dystrophic calcification in the placenta. Placental injury caused by the SARS-CoV-2 virus does not affect fetal Doppler parameters. It is not routinely necessary to use fetal Doppler in the obstetric follow-up of women who have SARS-CoV-2 infection during pregnancy and after recovery. However, fetal Doppler should be added to the examination process in the presence of other prenatal risks.

## References

[1] COVID-19 (2022). COVID-19 coronavirus pandemic.

[2] COVID-19 Treatment Guidelines (2023). Clinical spectrum of SARS-CoV-2 infection.

[3] Li M, Chen L, Zhang J, Xiong C, Li X (2020). The SARS-CoV-2 receptor ACE2 expression of maternal-fetal interface and fetal organs by single-cell transcriptome study. PLoS One.

[4] Schwartz DA, Morotti D, Beigi B, Moshfegh F, Zafaranloo N, Patanè L (2020). Confirming vertical fetal infection with coronavirus disease 2019: neonatal and pathology criteria for early onset and transplacental transmission of severe acute respiratory syndrome coronavirus 2 from infected pregnant mothers. Arch Pathol Lab Med.

[5] Fenizia C, Biasin M, Cetin I, Vergani P, Mileto D, Spinillo A (2020). Analysis of SARS-CoV-2 vertical transmission during pregnancy. Nat Commun.

[6] Shanes ED, Mithal LB, Otero S, Azad HA, Miller ES, Goldstein JA (2020). Placental pathology in COVID-19. Am J Clin Pathol.

[7] Girolamo R, Khalil A, Alameddine S, D’Angelo E, Galliani C, Matarrelli B (2021). Placental histopathology after SARS-CoV-2 infection in pregnancy: a systematic review and meta-analysis. Am J Obstet Gynecol MFM.

[8] Spinillo A, Gardella B, Bariselli S, Alfei A, Silini E, Bello B (2012). Placental histopathological correlates of umbilical artery Doppler velocimetry in pregnancies complicated by fetal growth restriction. Prenat Diagn.

[9] Salafia CM, Pezzullo JC, Minior VK, Divon MY (1997). Placental pathology of absent and reversed end-diastolic flow in growth-restricted fetuses. Obstet Gynecol.

[10] Jaiswal N, Puri M, Agarwal K, Singh S, Yadav R, Tiwary N (2021). COVID-19 as an independent risk factor for subclinical placental dysfunction. Eur J Obstet Gynecol Reprod Biol.

[11] Levitan D, London V, McLaren RA, Mann JD, Cheng K, Silver M (2021). Histologic and immunohistochemical evaluation of 65 placentas from women with polymerase chain reaction-proven severe acute respiratory syndrome coronavirus 2 (SARS-CoV-2) infection. Arch Pathol Lab Med.

[12] Glynn SM, Yang YJ, Thomas C, Friedlander RL, Cagino KA, Matthews KC (2022). SARS-CoV-2 and placental pathology: malperfusion patterns are dependent on timing of infection during pregnancy. Am J Surg Pathol.

[13] Curtin WM, Millington KA, Ibekwe TO, Ural SH (2017). Suspected fetal growth restriction at 37 weeks: a comparison of doppler and placental pathology. Biomed Res Int.

[14] Ayhan SG, Tanacan A, Atalay A, Sinaci S, Tokalioglu EO, Sahin D (2021). Assessment of fetal Doppler parameters in pregnant women with COVID-19 infection: a prospective case-control study. J Perinat Med.

[15] Soto-Torres E, Hernandez-Andrade E, Huntley E, Mendez-Figueroa H, Blackwell SC (2021). Ultrasound and Doppler findings in pregnant women with SARS-CoV-2 infection. Ultrasound Obstet Gynecol.

